# Etymologia: Meningococcal Disease

**DOI:** 10.3201/eid2307.ET2307

**Published:** 2017-07

**Authors:** Ronnie Henry

**Keywords:** meningococcal disease, Neisseria meningitidis, bacteria, meningitis/encephalitis, cerebrospinal fluid

## Meningococcal [mə-ningʺgo-kokʹal] Disease

From the Greek *meninx* (“membrane”) + *kokkos *(“berry”), meningococcal disease was first described by Vieusseux during an outbreak in Geneva in 1805. In 1884, Italian pathologists Ettore Marchiafava and Angelo Celli described intracellular micrococci in cerebrospinal fluid, and in 1887, Anton Wiechselbaum identified the meningococcus (designated as *Diplococcus intracellularis meningitidis*) in cerebrospinal fluid and established the connection between the organism and epidemic meningitis. Meningococcus can cause endemic cases, clusters, and epidemics of meningitis and septicemia (Figure).

**Figure:**
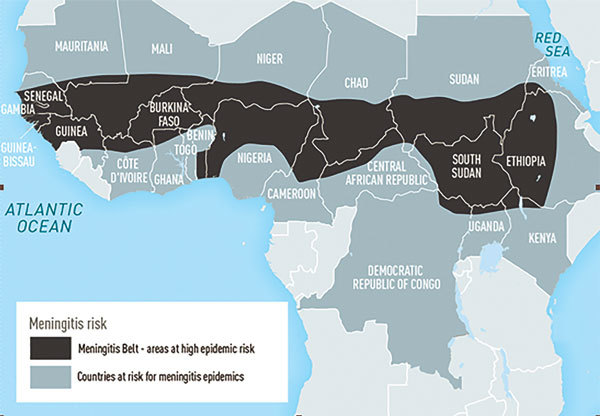
Areas with frequent epidemics of meningococcal meningitis. Data source:  World Health Organization, Geneva, Switzerland, 2012.

## References

[1] Apicella MA. *Neisseria meningitidis*. In: Mandell GL, Bennett, JE, Dolin R, editors. Mandell, Douglas, and Bennett’s Principles and Practice of Infectious Disease. 7th edition. Philadelphia: Elsevier; 2010. p. 2737–52.

[2] Manchanda V, Gupta S, Bhalla P. Meningococcal disease: history, epidemiology, pathogenesis, clinical manifestations, diagnosis, antimicrobial susceptibility and prevention. Indian J Med Microbiol. 2006;24:7–19. 10.4103/0255-0857.19888 16505549

[3] Stephens DS. Biology and pathogenesis of the evolutionarily successful, obligate human bacterium *Neisseria meningitidis.* Vaccine. 2009;27(Suppl 2):B71–7. 10.1016/j.vaccine.2009.04.070 19477055 PMC2712446

